# Supplementary figures and images for: Rhometa: Population recombination rate estimation from metagenomic read datasets
Source: PLoS Genet. 2023 Mar 27;19(3):e1010683. doi: 10.1371/journal.pgen.1010683 (PMC10079220; doi:10.1371/journal.pgen.1010683)

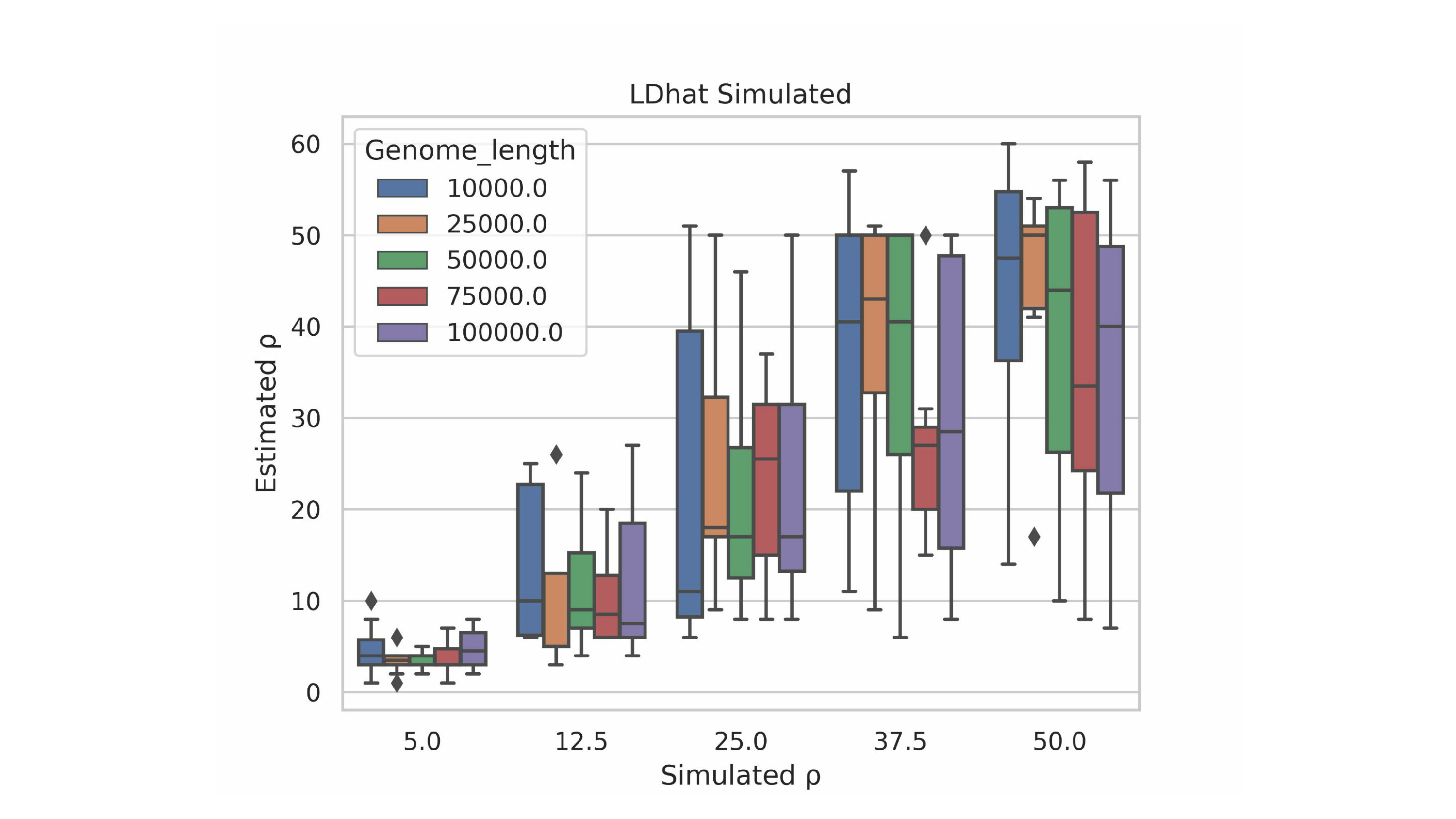

Supplement: S1 Fig — (TIF) [file pgen.1010683.s001.tif]

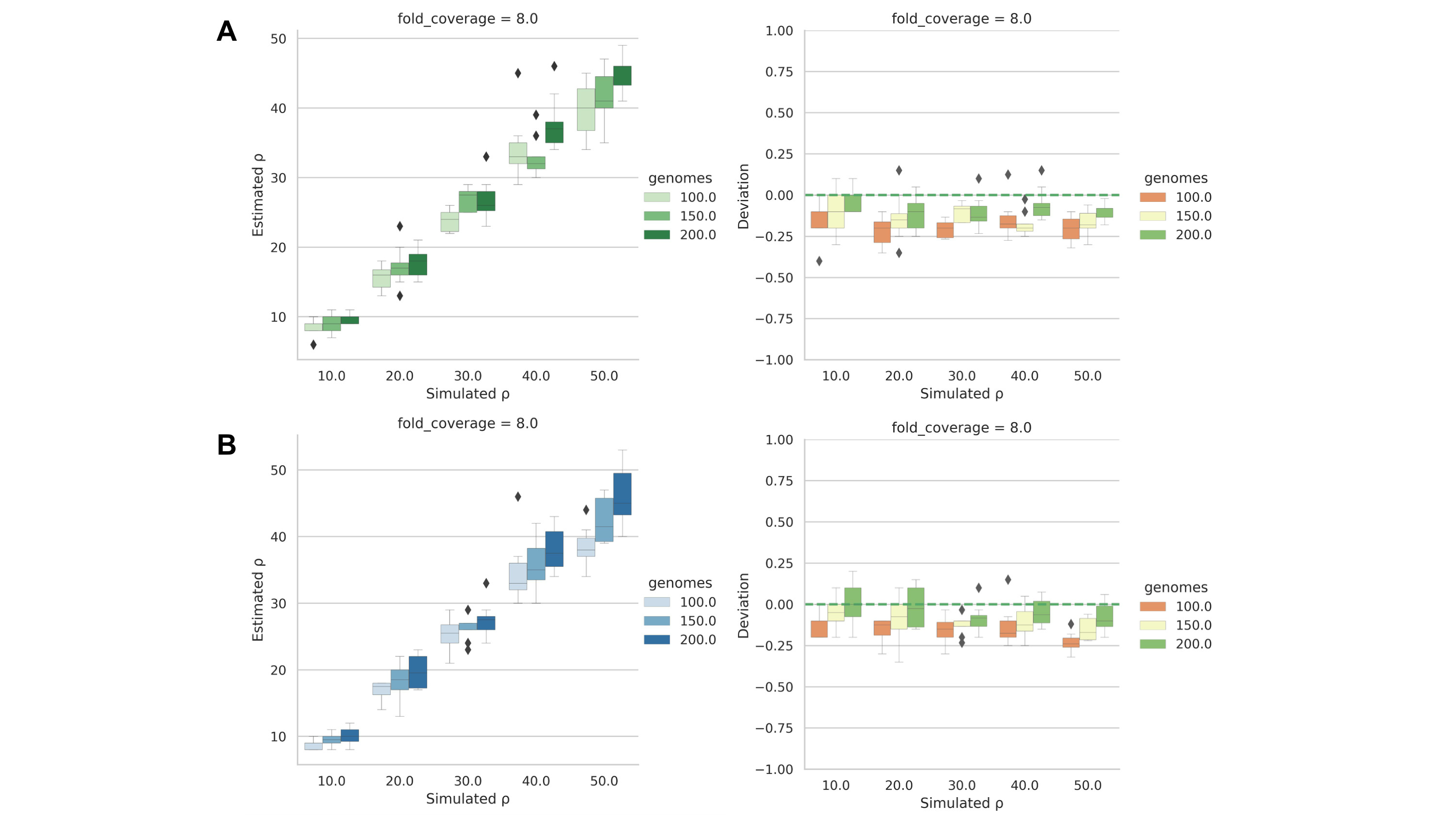

Supplement: S2 Fig — (A) Single end results. (B) Paired end results. (TIF) [file pgen.1010683.s002.tif]

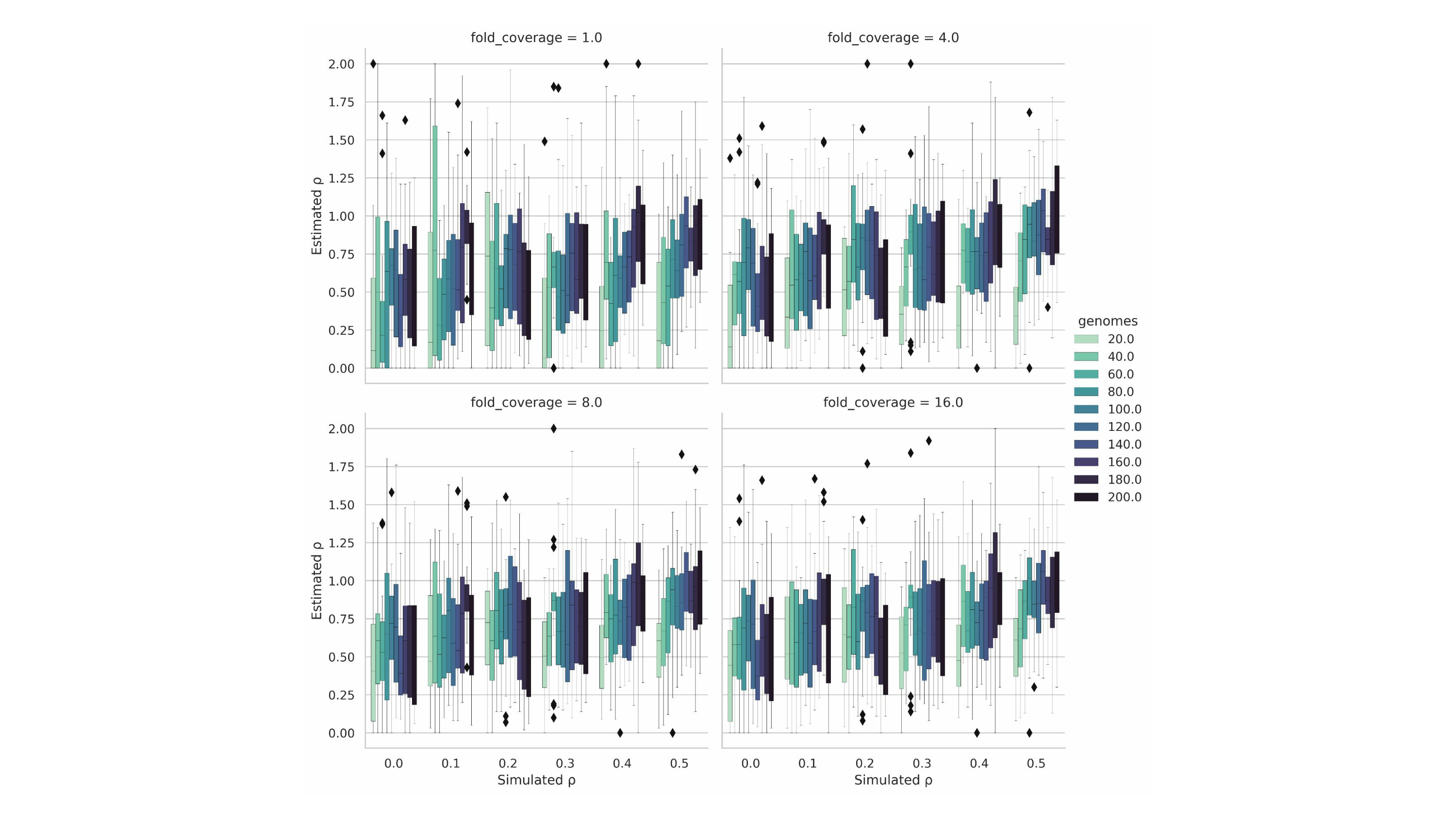

Supplement: S3 Fig — Results for varying numbers of simulated genomes and fold coverage values for population recombination rates 0.0, 0.1, 0.2, 0.3, 0.4, 0.5. The simulation parameters used are the same as for population recombination rates [10.0, 20.0, 30.0, 40.0, 50.0], except lookup tables for population recombination rates 0–2 were used (0–2 in 201 steps) for depths of 3–200. (TIF) [file pgen.1010683.s003.tif]

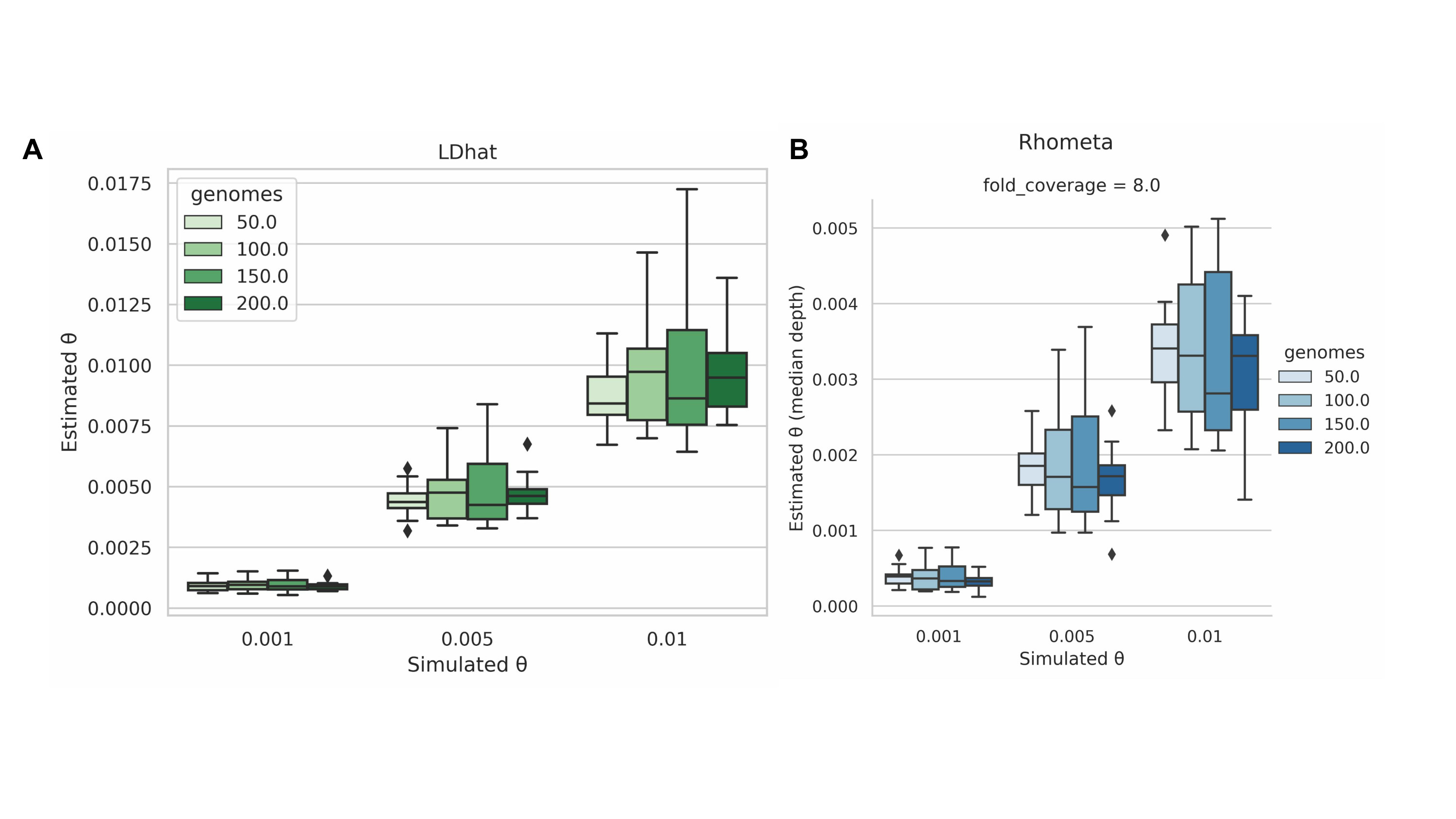

Supplement: S4 Fig — (A) LDhat. Simulated vs Estimated theta per site (θ) for varying number of simulated bacterial genomes. (B) Rhometa. Simulated vs Estimated theta per site (θ) for varying number of simulated bacterial genomes. (TIF) [file pgen.1010683.s004.tif]

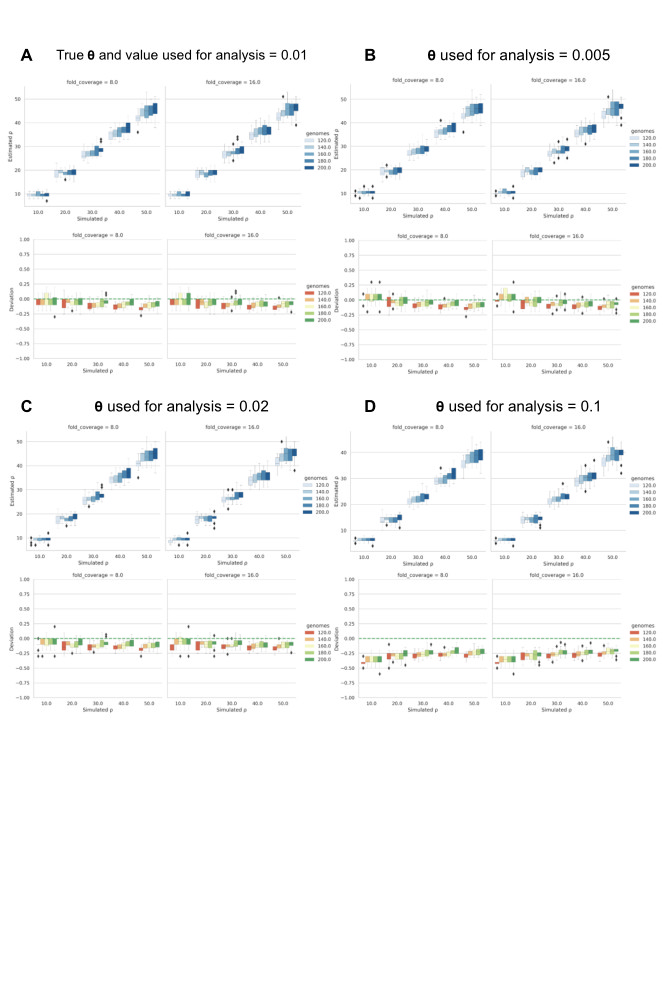

Supplement: S5 Fig — Results of analyzing simulated datasets with lookup tables generated under misspecified θ (genome length fixed at 100,000) (A) Simulated datasets analyzed with true θ (0.01) lookup tables (B) Simulated datasets analyzed with misspecified θ (0.005) lookup tables (C) Simulated datasets analyzed with misspecified θ (0.02) lookup tables (D) Simulated datasets analyzed with misspecified θ (0.1) lookup tables. (TIF) [file pgen.1010683.s005.tif]

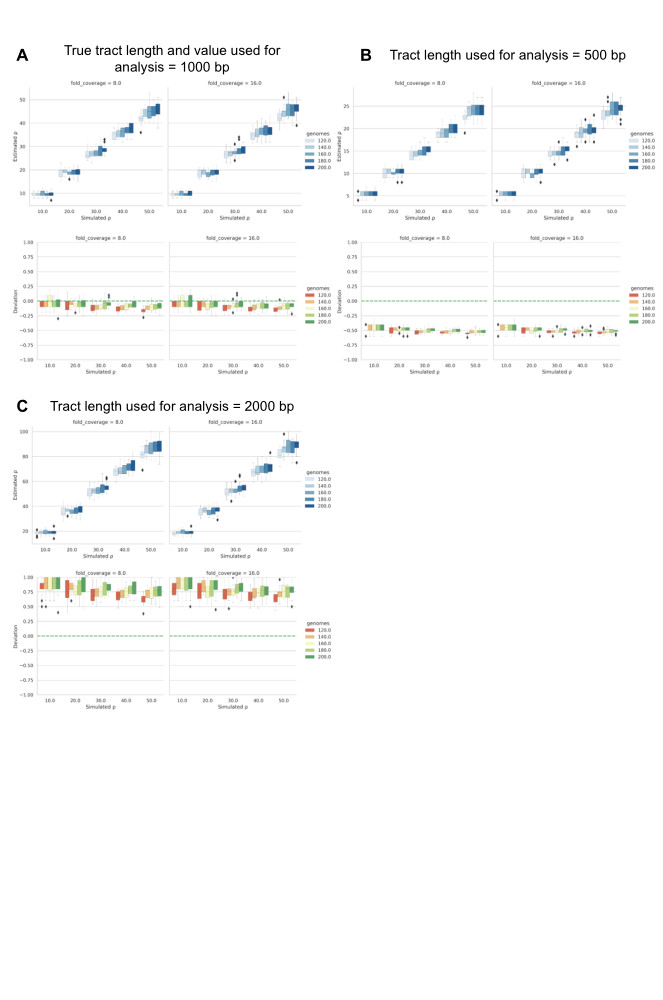

Supplement: S6 Fig — θ 0.01 lookup tables used) (A) Simulated datasets analyzed with true tract length 1000 bp (B) Simulated datasets analyzed with misspecified tract length 500 bp (C) Simulated datasets analyzed with misspecified tract length 2000 bp. (TIF) [file pgen.1010683.s006.tif]
