# Supplementary material for: Rhometa: Population recombination rate estimation from metagenomic read datasets
Source: PLoS Genet. 2023 Mar 27;19(3):e1010683. doi: 10.1371/journal.pgen.1010683 (PMC10079220; doi:10.1371/journal.pgen.1010683)
Supplement: S1 Table — Results for all seed values. (DOCX) [file pgen.1010683.s008.docx]

**S1 Table. Analysis results of s_pnemoniae transformation. Results for all seed values**

| Full sequence population recombination rate | | | |
| --- | --- | --- | --- |
| Seed value | exp-1 5ng | exp-1 500ng | 84 sequences |
| 0 | 6.8 | 4.4 | 4.7 |
| 1 | 7.7 | 6.4 | 5.8 |
| 2 | 3.4 | 4.9 | 3.7 |
| 3 | 5.7 | 7.7 | 4.9 |
| 4 | 4.2 | 2.7 | 3.3 |
| Mean | 5.56 | 5.22 | 4.48 |
| Per-site population recombination rate (Full seq/tract length) | | | |
| Seed value | exp-1 5ng | exp-1 500ng | 84 sequences |
| 0 | 0.00296 | 0.00191 | 0.00204 |
| 1 | 0.00335 | 0.00278 | 0.00252 |
| 2 | 0.00148 | 0.00213 | 0.00161 |
| 3 | 0.00248 | 0.00335 | 0.00213 |
| 4 | 0.00183 | 0.00117 | 0.00143 |
| Mean | 0.00242 | 0.00227 | 0.00195 |
| Tract length | 2300 |  |  |
