# Supplementary material for: Rhometa: Population recombination rate estimation from metagenomic read datasets
Source: PLoS Genet. 2023 Mar 27;19(3):e1010683. doi: 10.1371/journal.pgen.1010683 (PMC10079220; doi:10.1371/journal.pgen.1010683)
Supplement: S2 Table — (DOCX) [file pgen.1010683.s009.docx]

**S2 Table. *S.pneumoniae* experiment accession codes**

| **Strain** | **Experiment** | **Genotype** | **Treatment** | **Accession Code** |
| --- | --- | --- | --- | --- |
| 23F-R | - | Recipient | - | ERR016714 |
| TIGR4D*cps* | - | Donor | - | ERR028811 |
| TR1 | 1 | Wild type | Transformed with 5 ng mL^-1^ DNA | ERR025176 |
| TR2 | 1 | Wild type | Transformed with 5 ng mL^-1^ DNA | ERR025180 |
| TR3 | 1 | Wild type | Transformed with 5 ng mL^-1^ DNA | ERR025181 |
| TR4 | 1 | Wild type | Transformed with 5 ng mL^-1^ DNA | ERR025182 |
| TR5 | 1 | Wild type | Transformed with 5 ng mL^-1^ DNA | ERR025183 |
| TR7 | 1 | Wild type | Transformed with 5 ng mL^-1^ DNA | ERR025184 |
| TR8 | 1 | Wild type | Transformed with 5 ng mL^-1^ DNA | ERR025185 |
| TR9 | 1 | Wild type | Transformed with 5 ng mL^-1^ DNA | ERR025186 |
| TR10 | 1 | Wild type | Transformed with 5 ng mL^-1^ DNA | ERR025187 |
| TR11 | 1 | Wild type | Transformed with 5 ng mL^-1^ DNA | ERR025177 |
| TR12 | 1 | Wild type | Transformed with 5 ng mL^-1^ DNA | ERR025178 |
| TR13 | 1 | Wild type | Transformed with 5 ng mL^-1^ DNA | ERR025179 |
| TR14 | 1 | Wild type | Transformed with 5 ng mL^-1^ DNA | ERR025807 |
| TR15 | 1 | Wild type | Transformed with 5 ng mL^-1^ DNA | ERR025811 |
| TR17 | 1 | Wild type | Transformed with 5 ng mL^-1^ DNA | ERR025812 |
| TR18 | 1 | Wild type | Transformed with 5 ng mL^-1^ DNA | ERR025813 |
| TR19 | 1 | Wild type | Transformed with 5 ng mL^-1^ DNA | ERR025814 |
| TR20 | 1 | Wild type | Transformed with 5 ng mL^-1^ DNA | ERR025815 |
| TR22 | 1 | Wild type | Transformed with 5 ng mL^-1^ DNA | ERR025816 |
| TR24 | 1 | Wild type | Transformed with 5 ng mL^-1^ DNA | ERR025817 |
| TR25 | 1 | Wild type | Transformed with 5 ng mL^-1^ DNA | ERR025818 |
| TR26 | 1 | Wild type | Transformed with 5 ng mL^-1^ DNA | ERR025808 |
| TR27 | 1 | Wild type | Transformed with 5 ng mL^-1^ DNA | ERR025809 |
| TR29 | 1 | Wild type | Transformed with 5 ng mL^-1^ DNA | ERR025810 |
| TR31 | 1 | Wild type | Transformed with 5 ng mL^-1^ DNA | ERR025189 |
| TR32 | 1 | Wild type | Transformed with 5 ng mL^-1^ DNA | ERR025193 |
| TR33 | 1 | Wild type | Transformed with 5 ng mL^-1^ DNA | ERR025194 |
| TR34 | 1 | Wild type | Transformed with 5 ng mL^-1^ DNA | ERR025195 |
| TR35 | 1 | Wild type | Transformed with 5 ng mL^-1^ DNA | ERR025196 |
| TR36 | 1 | Wild type | Transformed with 5 ng mL^-1^ DNA | ERR025197 |
| TR37 | 1 | Wild type | Transformed with 5 ng mL^-1^ DNA | ERR025198 |
| TR38 | 1 | Wild type | Transformed with 5 ng mL^-1^ DNA | ERR025199 |
| TR39 | 1 | Wild type | Transformed with 5 ng mL^-1^ DNA | ERR025200 |
| TR42 | 1 | Wild type | Transformed with 5 ng mL^-1^ DNA | ERR025190 |
| TR43 | 1 | Wild type | Transformed with 5 ng mL^-1^ DNA | ERR025191 |
| TR44 | 1 | Wild type | Transformed with 5 ng mL^-1^ DNA | ERR025192 |
| TR45 | 1 | Wild type | Transformed with 5 ng mL^-1^ DNA | ERR025202 |
| TR46 | 1 | Wild type | Transformed with 5 ng mL^-1^ DNA | ERR025206 |
| TR47 | 1 | Wild type | Transformed with 5 ng mL^-1^ DNA | ERR025207 |
| TR48 | 1 | Wild type | Transformed with 5 ng mL^-1^ DNA | ERR025208 |
| TR49 | 1 | Wild type | Transformed with 5 ng mL^-1^ DNA | ERR025209 |
| TR50 | 1 | Wild type | Transformed with 500 ng mL^-1^ DNA | ERR025210 |
| TR51 | 1 | Wild type | Transformed with 500 ng mL^-1^ DNA | ERR025211 |
| TR52 | 1 | Wild type | Transformed with 500 ng mL^-1^ DNA | ERR025212 |
| TR53 | 1 | Wild type | Transformed with 500 ng mL^-1^ DNA | ERR025213 |
| TR54 | 1 | Wild type | Transformed with 500 ng mL^-1^ DNA | ERR025203 |
| TR55 | 1 | Wild type | Transformed with 500 ng mL^-1^ DNA | ERR025204 |
| TR56 | 1 | Wild type | Transformed with 500 ng mL^-1^ DNA | ERR025205 |
| TR57 | 1 | Wild type | Transformed with 500 ng mL^-1^ DNA | ERR025215 |
| TR60 | 1 | Wild type | Transformed with 500 ng mL^-1^ DNA | ERR025219 |
| TR61 | 1 | Wild type | Transformed with 500 ng mL^-1^ DNA | ERR025220 |
| TR62 | 1 | Wild type | Transformed with 500 ng mL^-1^ DNA | ERR025221 |
| TR63 | 1 | Wild type | Transformed with 500 ng mL^-1^ DNA | ERR025222 |
| TR64 | 1 | Wild type | Transformed with 500 ng mL^-1^ DNA | ERR025223 |
| TR65 | 1 | Wild type | Transformed with 500 ng mL^-1^ DNA | ERR025224 |
| TR66 | 1 | Wild type | Transformed with 500 ng mL^-1^ DNA | ERR025225 |
| TR68 | 1 | Wild type | Transformed with 500 ng mL^-1^ DNA | ERR025226 |
| TR69 | 1 | Wild type | Transformed with 500 ng mL^-1^ DNA | ERR025216 |
| TR70 | 1 | Wild type | Transformed with 500 ng mL^-1^ DNA | ERR025217 |
| TR71 | 1 | Wild type | Transformed with 500 ng mL^-1^ DNA | ERR025218 |
| TR72 | 1 | Wild type | Transformed with 500 ng mL^-1^ DNA | ERR025228 |
| TR73 | 1 | Wild type | Transformed with 500 ng mL^-1^ DNA | ERR025232 |
| TR74 | 1 | Wild type | Transformed with 500 ng mL^-1^ DNA | ERR025233 |
| TR76 | 1 | Wild type | Transformed with 500 ng mL^-1^ DNA | ERR025234 |
| TR77 | 1 | Wild type | Transformed with 500 ng mL^-1^ DNA | ERR025235 |
| TR78 | 1 | Wild type | Transformed with 500 ng mL^-1^ DNA | ERR025236 |
| TR79 | 1 | Wild type | Transformed with 500 ng mL^-1^ DNA | ERR025237 |
| TR80 | 1 | Wild type | Transformed with 500 ng mL^-1^ DNA | ERR025238 |
| TR81 | 1 | Wild type | Transformed with 500 ng mL^-1^ DNA | ERR025239 |
| TR83 | 1 | Wild type | Transformed with 500 ng mL^-1^ DNA | ERR025229 |
| TR85 | 1 | Wild type | Transformed with 500 ng mL^-1^ DNA | ERR025230 |
| TR86 | 1 | Wild type | Transformed with 500 ng mL^-1^ DNA | ERR025231 |
| TR87 | 1 | Wild type | Transformed with 500 ng mL^-1^ DNA | ERR025241 |
| TR88 | 1 | Wild type | Transformed with 500 ng mL^-1^ DNA | ERR025245 |
| TR89 | 1 | Wild type | Transformed with 500 ng mL^-1^ DNA | ERR025246 |
| TR90 | 1 | Wild type | Transformed with 500 ng mL^-1^ DNA | ERR025247 |
| TR91 | 1 | Wild type | Transformed with 500 ng mL^-1^ DNA | ERR025248 |
| TR93 | 1 | Wild type | Transformed with 500 ng mL^-1^ DNA | ERR025249 |
| TR94 | 1 | Wild type | Transformed with 500 ng mL^-1^ DNA | ERR025250 |
| TR95 | 1 | Wild type | Transformed with 500 ng mL^-1^ DNA | ERR025251 |
| TR96 | 1 | Wild type | Transformed with 500 ng mL^-1^ DNA | ERR025252 |
| TR98 | 1 | Wild type | Transformed with 500 ng mL^-1^ DNA | ERR025242 |
| TR99 | 1 | Wild type | Transformed with 500 ng mL^-1^ DNA | ERR025243 |
| TR100 | 1 | Wild type | Transformed with 500 ng mL^-1^ DNA | ERR025244 |
| SPWT1 | 2 | Wild type | Transformed with 500 ng mL^-1^ DNA | ERR052273 |
| SPWT2 | 2 | Wild type | Transformed with 500 ng mL^-1^ DNA | ERR052274 |
| SPWT3 | 2 | Wild type | Transformed with 500 ng mL^-1^ DNA | ERR052275 |
| SPWT4 | 2 | Wild type | Transformed with 500 ng mL^-1^ DNA | ERR052276 |
| SPWT5 | 2 | Wild type | Transformed with 500 ng mL^-1^ DNA | ERR052277 |
| SPWT6 | 2 | Wild type | Transformed with 500 ng mL^-1^ DNA | ERR052278 |
| SPWT7 | 2 | Wild type | Transformed with 500 ng mL^-1^ DNA | ERR052279 |
| SPWT8 | 2 | Wild type | Transformed with 500 ng mL^-1^ DNA | ERR052280 |
| SPWT9 | 2 | Wild type | Transformed with 500 ng mL^-1^ DNA | ERR052281 |
| SPWT10 | 2 | Wild type | Transformed with 500 ng mL^-1^ DNA | ERR052282 |
| SPWT11 | 2 | Wild type | Transformed with 500 ng mL^-1^ DNA | ERR052283 |
| SPWT12 | 2 | Wild type | Transformed with 500 ng mL^-1^ DNA | ERR052284 |
| SPWT13 | 2 | Wild type | Transformed with 500 ng mL^-1^ DNA | ERR052285 |
| SPWT14 | 2 | Wild type | Transformed with 500 ng mL^-1^ DNA | ERR052286 |
| SPWT16 | 2 | Wild type | Transformed with 500 ng mL^-1^ DNA | ERR052288 |
| SPWT18 | 2 | Wild type | Transformed with 500 ng mL^-1^ DNA | ERR052290 |
| SPWT19 | 2 | Wild type | Transformed with 500 ng mL^-1^ DNA | ERR052291 |
| SPWT20 | 2 | Wild type | Transformed with 500 ng mL^-1^ DNA | ERR052292 |
| SPWT21 | 2 | Wild type | Transformed with 500 ng mL^-1^ DNA | ERR052293 |
| SPWT24 | 2 | Wild type | Transformed with 500 ng mL^-1^ DNA | ERR052296 |
| SPHB1 | 2 | D*hexB* | Transformed with 500 ng mL^-1^ DNA | ERR052249 |
| SPHB2 | 2 | D*hexB* | Transformed with 500 ng mL^-1^ DNA | ERR052250 |
| SPHB4 | 2 | D*hexB* | Transformed with 500 ng mL^-1^ DNA | ERR052252 |
| SPHB5 | 2 | D*hexB* | Transformed with 500 ng mL^-1^ DNA | ERR052253 |
| SPHB7 | 2 | D*hexB* | Transformed with 500 ng mL^-1^ DNA | ERR052255 |
| SPHB8 | 2 | D*hexB* | Transformed with 500 ng mL^-1^ DNA | ERR052256 |
| SPHB9 | 2 | D*hexB* | Transformed with 500 ng mL^-1^ DNA | ERR052257 |
| SPHB10 | 2 | D*hexB* | Transformed with 500 ng mL^-1^ DNA | ERR052258 |
| SPHB11 | 2 | D*hexB* | Transformed with 500 ng mL^-1^ DNA | ERR052259 |
| SPHB12 | 2 | D*hexB* | Transformed with 500 ng mL^-1^ DNA | ERR052260 |
| SPHB13 | 2 | D*hexB* | Transformed with 500 ng mL^-1^ DNA | ERR052261 |
| SPHB14 | 2 | D*hexB* | Transformed with 500 ng mL^-1^ DNA | ERR052262 |
| SPHB15 | 2 | D*hexB* | Transformed with 500 ng mL^-1^ DNA | ERR052263 |
| SPHB17 | 2 | D*hexB* | Transformed with 500 ng mL^-1^ DNA | ERR052265 |
| SPHB18 | 2 | D*hexB* | Transformed with 500 ng mL^-1^ DNA | ERR052266 |
| SPHB19 | 2 | D*hexB* | Transformed with 500 ng mL^-1^ DNA | ERR052267 |
| SPHB20 | 2 | D*hexB* | Transformed with 500 ng mL^-1^ DNA | ERR052268 |
| SPHB22 | 2 | D*hexB* | Transformed with 500 ng mL^-1^ DNA | ERR052270 |
| SPHB23 | 2 | D*hexB* | Transformed with 500 ng mL^-1^ DNA | ERR052271 |
| SPHB24 | 2 | D*hexB* | Transformed with 500 ng mL^-1^ DNA | ERR052272 |
