# Supplementary material for: Rhometa: Population recombination rate estimation from metagenomic read datasets
Source: PLoS Genet. 2023 Mar 27;19(3):e1010683. doi: 10.1371/journal.pgen.1010683 (PMC10079220; doi:10.1371/journal.pgen.1010683)
Supplement: S3 Table — (DOCX) [file pgen.1010683.s010.docx]

**S3 Table. Accession codes for Port Hacking, Sydney datasets (from SRA run table)**

| **Run** | **BioProject** | **BioSampleModel** | **Collection_Date** | **Sample Name** | **Lat_Lon** |
| --- | --- | --- | --- | --- | --- |
| SRR7609609 | PRJNA481422 | Metagenome or environmental | 2013-05 | Port Hacking TJ14 | 34.1192 S 151.2267 E |
| SRR7609610 | PRJNA481422 | Metagenome or environmental | 2013-06 | Port Hacking TJ15 | 34.1192 S 151.2267 E |
| SRR7609611 | PRJNA481422 | Metagenome or environmental | 2013-09 | Port Hacking TJ18 | 34.1192 S 151.2267 E |
| SRR7609612 | PRJNA481422 | Metagenome or environmental | 2013-08 | Port Hacking TJ17 | 34.1192 S 151.2267 E |
| SRR7609613 | PRJNA481422 | Metagenome or environmental | 2012-08 | Port Hacking TJ5 | 34.1192 S 151.2267 E |
| SRR7609614 | PRJNA481422 | Metagenome or environmental | 2012-09 | Port Hacking TJ6 | 34.1192 S 151.2267 E |
| SRR7609615 | PRJNA481422 | Metagenome or environmental | 2012-10 | Port Hacking TJ7 | 34.1192 S 151.2267 E |
| SRR7609616 | PRJNA481422 | Metagenome or environmental | 2012-11 | Port Hacking TJ8 | 34.1192 S 151.2267 E |
| SRR7609617 | PRJNA481422 | Metagenome or environmental | 2013-01 | Port Hacking TJ10 | 34.1192 S 151.2267 E |
| SRR7609618 | PRJNA481422 | Metagenome or environmental | 2013-02 | Port Hacking TJ11 | 34.1192 S 151.2267 E |
| SRR7609619 | PRJNA481422 | Metagenome or environmental | 2013-03 | Port Hacking TJ12 | 34.1192 S 151.2267 E |
| SRR7609620 | PRJNA481422 | Metagenome or environmental | 2013-04 | Port Hacking TJ13 | 34.1192 S 151.2267 E |
| SRR7609621 | PRJNA481422 | Metagenome or environmental | 2013-07 | Port Hacking TJ16 | 34.1192 S 151.2267 E |
| SRR7609622 | PRJNA481422 | Metagenome or environmental | 2012-12 | Port Hacking TJ9 | 34.1192 S 151.2267 E |
| SRR7609623 | PRJNA481422 | Metagenome or environmental | 2012-07 | Port Hacking TJ4 | 34.1192 S 151.2267 E |
