# Supplementary material for: Rhometa: Population recombination rate estimation from metagenomic read datasets
Source: PLoS Genet. 2023 Mar 27;19(3):e1010683. doi: 10.1371/journal.pgen.1010683 (PMC10079220; doi:10.1371/journal.pgen.1010683)
Supplement: S1 Appendix — Matching against the lookup table. (DOCX) [file pgen.1010683.s011.docx]

# S1 Appendix

## Lookup configuration

Using the lookup tables requires converting the bi-allelic variant site pairs into a different configuration, which we call the “lookup configuration”. For this step we made use of some functions from Pyrho, where the input has to be in a particular manner, we will explain the LDhat approach first, then the change made for Pyrho.

Let’s consider an earlier example with bi-allelic variant site pair (4,9) with 3 CA and 2 GT as bases, we first need to identify the major allele denoted by 0 and minor allele denoted by 1. For the first site, variant site 4 we have 3 Cs and 2Gs so the major allele (0) is C and the minor allele (1) is G, likewise for the second site variant site 9 we have 3 As and 2 Ts so 0 is A and 1 is T. The lookup configuration is in the format {00, 01, 10, 11}, 00 is site 1 major allele and site 2 major allele, 01 is site 1 major allele and site 2 minor allele and so on.

For 00, the major allele for site 1 is C and the major allele for site 2 is A. Next we look at how many CA pairs there are, we have 3. For 01 we would consider CT and see how many CT pairs we have, which is 0. Continuing in this manner we get the following final results for our bi-allelic pairs, 3 CAs and 2 GTs (CA, CA, CA, GT, GT) becomes {00 : 3, 01 : 0, 10 : 0, 11 : 2}.

## Matching against the lookup table

The index of the lookup tables is in the format {00, 01, 10, 11} by converting the bases for the variant sites into this format we can match against the lookup table and get the likelihoods for each variant site pair, this is done for the entire bi-allelic filtered pairwise table. We used some Pyrho functions for this step, this was done to avoid unnecessary code rewrites and because there are complexities involved in implementation, for instance there needs to be a method in place for handling cases where there is no clear major and minor allele for a site pair i.e. there are a even number of alleles, such as a site pair position with 2 AA and 2 TT. Pyrho, specifically their rho_splines.py script and the compute_splines method therein, has an excellent approach to this step, which we have incorporated into our program. The pyro approach, however, requires the input be formatted in a specific manner.

For the Pyrho method, which is quite a bit simpler, let's again consider our example with bi-allelic variant site pair (4,9) with 3 CA and 2 GT as bases. First we consider the unique bases at each variant site, for site 1 (variant site 4) we have C and G and for site 2 (variant site 9) we have A and T. For the next step Pyrho encodes the nucleotide bases in this manner { A : 2, C : 3, G : 4, T : 5 }, so for site 1 we have C : 3 and G : 4 and for site 2 we have A : 2 and T : 5. We then use the number associated to the base to determine the major and minor allele per site, for site 1 in G with associated number 4 is the major allele (0) since it is greater than C with associated number 3 which is the minor allele (1), likewise the major and minor alleles for site 2 are T and A respectively.

Having identified the major and minor allele for site 1 and site 2, this needs to be converted in the lookup configuration {00, 01, 10, 11} using the method described earlier the major alleles for site 1 and site 2 are G and T respectively, which is 00, we have 2 GTs so the value for 00 is 2, continuing in this manner we get { 00: 2, 01 : 0 , 10 : 0, 11 : 3}. This process is applied to the entire bi-allelic pairwise table, then the lookup configurations from this table along with an appropriate size lookup table can be used by Pyrho to perform the matching and get the likelihoods for the variant site pairs.
