# Supplementary material for: Rhometa: Population recombination rate estimation from metagenomic read datasets
Source: PLoS Genet. 2023 Mar 27;19(3):e1010683. doi: 10.1371/journal.pgen.1010683 (PMC10079220; doi:10.1371/journal.pgen.1010683)
Supplement: Attachment — Submitted filename: Reviewer Feedback Response .pdf [file pgen.1010683.s012.pdf]

UTS UNIVERSITY OF TECHNOLOGY SYDNEY

Sidaswar Krishnan
PhD Student

Climate Change Cluster, Faculty of Science
15 Broadway, Ultimo NSW 2007

sidaswar.krishnan-1@student.uts.edu.au

PO Box 123
Broadway
NSW 2007 Australia
www.uts.edu.au

UTS CRICOS PROVIDER CODE 00099F

Dear Dr Xavier Didelot and Dr Xiaofeng Zhu,

We thank you for handling our manuscript “Rhometa: Population recombination rate estimation from metagenomic read datasets“ (PGENETICS-D-22-00986). We wish to thank the reviewers for their valuable feedback, which we believe has helped us to significantly improve the manuscript. Many of the additions and changes bring further clarity to what we hope to accomplish and contribute with this new program.

As requested by the reviewers, we have performed further evaluation of Rhometa using simulated datasets. We show that Rhometa is robust to misspecifications of θ and that misspecifying the tract length tends to linearly scale the final estimates in a predictable manner. Additionally we applied the simulated datasets for recombination rate estimation, used on Rhometa, to mcorr and demonstrated that Rhometa performs significantly better.

Furthermore, in-line with the reviewer’s requests, we have now applied Rhometa to real metagenomic datasets, using samples from an ocean environment in eastern Australia and demonstrate that Rhometa does indeed work well on real metagenomic datasets.

Please find our detailed response to each of the reviewer’s comments below. We hope that with these new additions and changes that you feel the manuscript is now suitable for publication.

Yours sincerely,

Sidaswar Krishnan

Reviewers' comments:

For ease of editorial review, we have included the Reviewers' comments below in bold. Our responses are in *italics and in blue*. **Text that has been inserted or changed within the modified manuscript is** underlined. **Line numbers follow marked-up copy (PDF).**

Reviewer #1: In this article, the authors have introduced a method to infer the population recombination rate in prokaryotic populations, which is novel and can be very useful in a bunch of analysis. The authors built up a pipeline of going from metagenomic data of reads to vcf files to plug into the framework of LDpop, with a gene-conversion based recombination model. In brief, I think the method and analysis in this article is really solid and the software can be really promising in a lot of studies. Here are some of my minor comments to this manuscript:

1.1 In line 123 you said that pyrho is a method supporting read-based data but is only workable for diploid genomes. However, as far as I understand, the basic methodology of pyrho is not that different from LDhat or LDpop except that pyrho can handle a population size history and I believe the input format needs to be a vcf file instead of reads data directly. I recommend correcting the mention of pyrho here (if what I recall is correct), and really mention the contribution of your work on handling the bioinformatics of read data and the modules in the pipeline.

We would like to thank the reviewer for their feedback. Regarding LDpop, it is only able to generate lookup tables and cannot be used on its own for recombination rate estimation. The mathematical formulations for haploid and diploid are different.

Regarding pyRho and Rhometa, pyRho only supports diploid genomes which experience recombination in form of crossing-over and thus cannot be used with prokaryote genomes where recombination is of the gene-conversion type. The methods for handling crossing-over and gene-conversion are mathematically distinct. LDhat has methodologies implemented for both types and provides options for selecting the different methods.

1.2 I might have missed this but you mentioned using the approx option while generating the lookup table from LDpop, did you benchmark the performance of inference when generating the table using approx, especially for larger sample size? It would be nice to see some simulation-based benchmarking results while having a large sample size and using approx option to give people a sense of how much accuracy is lost by doing this.

For Rhometa using the exact lookup table generation is not tractable. We need to generate lookup tables with large sample sizes, which uses a lot of computational resources. As such we don't provide this option in our lookup table generation pipeline by default. As the authors of LDpop have stated on the program LDpop github page (https://github.com/popgenmethods/ldpop) the approx option provides a "reasonable approximation that is much faster and scales to larger sample sizes"

The following line has been added under the Lookup tables section (Line 278). “This also makes generation of large lookup tables, as is necessary with Rhometa, more tractable”.

1.3 Did you investigate sequencing errors while simulating reads? Sequencing errors can give false signals to recombinations and thus confound the analysis. I would suggest investigating the robustness of the method under different error rates (close to real world sequencing technology parameters).

Yes, we indeed simulated reads with sequencing errors using the ART program. To clarify this in the manuscript, the following line has been added to the main text (Line 489): “ ART [35], which simulates sequencing reads by mimicking real sequencing processes with empirical error models. The reads were simulated based on the illumina HiSeqX TruSeq system”

Overall I think this work is really solid and the authors have made good points in the use of the method. I hope this could contribute to the field and facilitate a lot of studies!

We thank the reviewer for their positive appraisal.

Reviewer #2: Review of "Rhometa: Population recombination rate estimation from metagenomic read datasets"

This paper introduces a new computational pipeline for estimating recombination rates in metagenomic datasets. The overall goal is very important - as the authors emphasise, the majority of microorganisms cannot be cultured and hence we only observe them in in their natural, complex, environments via metagenomics. The software pipeline has been well explained and care has been taken to ensure that it is appropriate and can run at scale.

Unfortunately I have concerns with the paper as it stands:

2.1 Firstly, the writing is far from the expected standards. I've described many specifics below but overall it needs a complete and thorough overhaul from an experienced scientific writer. In places this gets in the way of review as I could not quite follow the details. Except in the limitations section (which seemed strong), there was little in the way of reflection or consideration about the details, and the text needs to provide more insight and less technical repetition.

In response to the reviewer's comments, we have strived to raise the standard of our writing. We extend our thanks to the reviewer for pointing out the specific changes needed. We have taken the reviewer's suggestions into account and made the necessary revisions to the manuscript. We believe that the improvements made as a result of the reviewer's suggestions have greatly elevated the quality of the manuscript.

2.2 To give an example from the abstract: "the deviation from the expected value decreased as the number of genomes increased" is about all that is claimed, but this is just one minor pre-requisite for all publishable methods... What can we say that is meaningful about its performance? How does it compare to the state of the art? Does it do something that is currently impossible otherwise? The same writing problem exists throughout.

We thank the reviewer for their feedback, the text has been revised to read (Line 35): "Rhometa offers a comprehensive solution for determining population recombination rates from contemporary metagenomic read datasets. Rhometa extends the capabilities of conventional sequence-based composite likelihood population recombination rate estimators to include modern aligned metagenomic read datasets with diverse sequencing depths, thereby enabling the effective application of these techniques and their high accuracy rates to the field of metagenomics."

2.3 On Line 621 it is explained that the essence of this paper is that "for aligned read based datasets the main complication is that the depth can vary greatly from site to site." This is conceptually very straight forward, and just requires (a lot of!) careful technical work to implement (for great value, I agree). That intuition should come through more cleanly throughout (starting in the abstract).

We thank the reviewer for this suggestion. In response we have now:

Added the following paragraph to the abstract (Line 36): "Rhometa extends the capabilities of conventional sequence-based composite likelihood population recombination rate estimators to include modern aligned metagenomic read datasets with diverse sequencing depths, thereby enabling the effective application of these techniques and their high accuracy rates to the field of metagenomics."

In addition, the section which started at line 621 has now been updated to (Line 1020): "A complication associated with read-based datasets is that the depth can vary greatly from site to site. We addressed this issue by using an appropriate depth lookup table for the variant site pairs being considered, the rationale for which is that the negative log-likelihoods for the variant site pairs considered is obtained individually and then combined for a final composite negative log-likelihood. Taking into account that the negative log-likelihoods are obtained individually for each pair of variant sites, the variant pair combinations considered are limited to the extent of a read for single-end reads, for the insert length in the case of pair-end reads.

This provides a way for us to group the variant sites by depth, following which we use the appropriate depth negative log-likelihood table to obtain the negative log-likelihoods for each pair and then finally combine the negative log-likelihoods to get a result for the entire dataset. Additionally, we have introduced a novel weighted sum when calculating the composite negative log-likelihood across coverage depth (equation 2). Rhometa thus enables the application of the composite likelihood estimator approach for current shotgun metagenomic datasets."

We have also made numerous minor changes throughout the manuscript to enhance the clarity of the text

2.4 Secondly, I did not find the analysis of the data to be completely compelling. Results are only given for "rho", the population recombination rate, in one simulation scenario (varying true rho and data quantity). I would have expected some sensitivity analysis around critical parameters - the average tract length, but also population demography, variation of rho along the genome, presence of related strains, etc.

In response to the reviewer's request, we have now performed tract length and theta simulation sweeps. The results of which are addressed in the discussion, limitations and future directions section. The new content added related to tract length and theta sweeps respectively are as follows:

(Line 1070)“In our tests with simulated datasets where the tract length was varied, it was found that misspecifying the tract length tends to linearly scale the final estimates in a predictable manner (S6 Fig). If a fixed value of tract length is given by the user for all analyses (i.e. an accurate sample-specific tract length is not provided by the user), then differences in inferred rho among samples could be due to either a change in the recombination rate or a change in tract length. The user will need to take this into account when interpreting the results.For instance, if performing same species comparisons across time and location, the tract length can be fixed to determine relative changes in the magnitude of recombination.”

(Line 1095 “Generation of lookup tables require specification of θ *, in our tests we have found that Rhometa is fairly robust to misspecifications of* θ*. It is only extreme misspecifications, such as misspecifications by a factor of 10, that seem to have a large impact (S5 fig).”*

Rhometa is an important first step for inferring the recombination rate from metagenomic reads. Factoring in population demography/ effective population size is a complicated issue. Also it is difficult to infer population demography/ effective population size in metagenomics.

Additionally the combinatorial complexity will be too high to factor in the impact of all the variables mentioned and their interactions. These can be a future area of research.

2.5 I'd like head to head comparisons where this is possible. However, since this is arguably the first method to do what it does, it doesn't matter if some of the experiments fail - but this information is critical to users for interpretation. (the slight bias in the inference for high rho is a case in point.)

In response to the reviewer's request, the following paragraphs, looks at critical parameters and their impact on final ρ *estimates. These important parameters are user adjustable and this new information can help assist the user in determining which values to set:*

(Line 1070) “In our tests with simulated datasets where the tract length was varied, it was found that misspecifying the tract length tends to linearly scale the final estimates in a predictable manner (S6 Fig). If a fixed value of tract length is given by the user for all analyses (i.e. an accurate sample-specific tract length is not provided by the user), then differences in inferred rho among samples could be due to either a change in the recombination rate or a change in tract length. The user will need to take this into account when interpreting the results.For instance, if performing same species comparisons across time and location, the tract length can be fixed to determine relative changes in the magnitude of recombination.”

(Line 1095) “Generation of lookup tables require specification of θ *, in our tests we have found that Rhometa is fairly robust to misspecifications of* θ*. It is only extreme*

misspecifications, such as misspecifications by a factor of 10, that seem to have a large impact (S5 fig).”

2.6 I did not find the real data analysis convincing to the central claim that the method was better than its rivals, but that may be because I could not follow the exposition around it. Details are below.

To address this concern, we have now performed an additional simulation study with mcorr, where the simulated datasets used for Rhometa was also used with mcorr. The following paragraph has been added to the discussion which addresses these results:

(Line 905) “Furthermore, mcorr was evaluated on simulated datasets used to test for Rhometa (S7 fig) where we expected to see an increase in the ϕ_pool estimated as the simulated ρ increases. However, the results showed no correlation between the recombination rate inferred by mcorr and the true simulated rate. Additionally, ϕ_pool, the value for recombination estimated by mcorr complicates interpretability by not using the standard parameter for population recombination (ρ). mcorr’s limitation of looking at coding regions provided in a gene annotation file again complicates analysis as this information is not always available. Rhometa builds on established methods and theoretical foundations, without the need for gene annotation files and based on our testing, produces accurate results.”

This is not a criticism, but I am unsure what to say about the novelty of this work. What novelty there is here is in putting together work into a well functioning pipeline to solve a particular scientific need. I would like to support the paper against such a criticism because I do think there is huge value in making these tools well from a software engineering perspective, and having venues for them to be published.

We thank the reviewer for their support and agree.

2.7 Finally I'd like to see something about computational requirements, for users to get a scale for when this is practical.

In response to this request, the following 3 lines have added to the manuscript, referring to computational resources:

(Line 74) “The program performs well on large BAM files and the included subsampling functionality ensures that files of arbitrarily large size are subsampled and analysed.” Talks about how very large files can be processed.

(Line 279) Generally, the generation of large lookup tables is a computationally intensive process, more information on which can be found in the paper associated with LDpop [32].

(Line 824) The 84 sequence dataset was also used to benchmark Rhometa. The bam file for this dataset is 11.42 GB in size. On a system with 4 CPUs and 32 GB RAM the rho_est pipeline took 1h 45m to complete and the theta_est pipeline took 2h 45m to complete.

Details and minor points:

2.8 Abstract "In simulated datasets, the deviation from the expected value decreased as the number of genomes increased " is hardly a headline result. I suggest replacing it with something more specific.

As suggested by the reviewer, this text has now been modified to read (Line 35): "Rhometa offers a comprehensive solution for determining population recombination rates from contemporary metagenomic read datasets. Rhometa extends the capabilities of conventional sequence-based composite likelihood population recombination rate estimators to include modern aligned metagenomic read datasets with diverse sequencing depths, thereby enabling the effective application of these techniques and their high accuracy rates to the field of metagenomics."

2.9 Summary /Abstract: say something about the scale of data your method is suitable for.

In the author summary I have added the following paragraph to provide more information on this point (Line 74): The program performs well on large BAM files and the included subsampling functionality ensures that files of arbitrarily large size are subsampled and analysed.

Additionally I have updated the lookup table and depth section in methods to provide more details. The updated paragraph is as follows (Line 301): Where the bam file is of a high depth, this can result in pairwise subtables of high depth. In such cases, available lookup tables may not be of high enough depth to cover them. A subsampling feature is included that is able to automatically downsample the BAM to a given depth.

2.10 Line 87,89: I found the final units unclear "rate of initiation of gene conversion" and "mutation rate". Indeed the line below confuses the matter as we then divide by the mutation rate.

We have now changed wording to recombination rate to avoid confusion.

2.11 I did not find the separation of the mcorr method convincing. The description is confused and doesn't make it clear exactly what mcorr is estimating and why we would prefer what Rhometa does.

To address this point we have updated the discussion as follows (Line 904): Meanwhile, with mcorr, the mathematical formulation is distinct from the well-known population recombination rate ($\rho = 2N_e r$), which may represent challenges for interpretation. On an experimental dataset where transformation was used to produce a population of recombinants for sequencing, the approach implemented in mcorr appears to significantly underestimate the recombination rate. Furthermore, mcorr was evaluated on simulated datasets used to test Rhometa (S7 fig) where we expected to see an increase in the ϕ_pool estimated as the as the simulated ρ increases, however, and the results showed no correlation between the recombination rate inferred by mcorr and the true simulated rate. Additionally, ϕ_pool, the value for recombination estimated by mcorr complicates interpretability by not using the standard parameter for population recombination (ρ). mcorr's limitation of looking at coding regions provided in a gene annotation file again complicates analysis as this information is not always available. Rhometa builds on established methods and theoretical foundations.

without the need for gene annotation files and based on our testing, produces accurate results."

Additionally, some small changes have also been made to the mcorr section in the introduction, taken together we feel it builds a strong case for why Rhometa is better for metagenomic datasets.

2.12 Line 153: "We have carefully adapted the program to work with modern read based metagenomic datasets." Elsewhere it is implied that this is new software, and I think it probably is? Be very clear about where the code is coming from. (It is natural that the ideas behind LDhat would be incorporated, and this is and should be acknowledged.)

As per the reviewer's suggestion the section has been updated as follows (Line 193): "Our approach focuses on advancing the composite likelihood approach for use with aligned metagenomic read datasets. We have built our metagenomic population recombination rate estimator program upon the approach introduced in the LDhat program, specifically the LDhat pairwise module [12]. LDhat is a well known and used program with support for microbial datasets, specifically for the gene-conversion type recombination which occurs in microbes, however, it is limited to aligned genome sequences. We have designed Rhometa to work with aligned read based metagenomic datasets, where the complication of varying depths and short reads needs to be addressed."

2.13 Watterson's Theta estimate will be biased after accounting for alignment and recombination, compared to the estimate of theta that would be obtained by a likelihood-based approach (which could be the "true parameter value" from some model). Typically this would be handled by an iterative algorithm and usually does not take many iterations? At least the bias needs discussing.

The reviewer raises a valid concern. The Watterson's theta estimate implemented here follows the approach used in the LDhat program, which is not likelihood based. The composite likelihood approach and the likelihood tables are only used for ρ. We chose to stay close to the Waterson estimate formulation as it is well established and tested and we did not want to make significant changes to it here. It takes it into account all variant sites, be it due to mutation or recombination for estimate by default, it only matters that it is a variant site. Which could cause it to be biased towards higher rates of mutation as the reviewer suggested. Clearly referring back to the original LDhat paper and the formulation and explanation described therein should make it clear to the reader its limitations and potential biases.

Alongside variant sites, it takes into account the genome length and number of sequences, however since for a metagenomic we don't know the exact number of genomes/sequences, we have instead used the mean and median depth though the true number of different genomes in a metagenomic sample is likely to be significantly higher, as such using the depth value is reasonable and in many cases is likely to be a conservative estimate for the number of genomes.

We have made clear our implementation closely follows the LDhat implementation, knowing its derivation should make it clear to the reader its advantages and limitations and also further explained why we used depth in place of genomes (Line 283):

"A subprogram is provided to estimate the population mutation rate ($\theta = 2N_e u$), per site. The formulation is directly based on Watterson's theta estimate as implemented in LDhat [12] [12], with some changes on the information used for the input parameter. The program requires the aligned BAM file and the reference FASTA file and makes use of freebayes to identify variant sites which is required for the Watterson estimate. θ is a required parameter for lookup table generation adjusted for read based datasets, the θ estimate is calculated based on dataset depth – specifically mean and median depth – in place of the number of genomes, as in the original formation (S4 Fig). For metagenomic where the exact number of genomes is unknown, the true number of different genomes in a metagenomic sample is likely to be a large value, as such using the depth value is reasonable and in many cases is likely to be a conservative estimate for the number of genomes. "

Our primary goal with this paper is to estimate the recombination rate, to this end we demonstrated that the ρ estimation pipeline is robust to misspecifications in θ in the following new paragraph added to the discussion:

(Line 1095) "Generation of lookup tables require specification of θ , in our tests we have found that Rhometa is fairly robust to misspecifications of θ. It is only extreme misspecifications, such as misspecifications by a factor of 10, that seem to have a large impact (S5 fig)."

2.14 The section "Lookup table and depth" is very fuzzy. What specifically do you do? It sounds like there is a threshold (minimum depth?) to which you subsample down to? Or is this entirely optional?

We apologize for the lack of clarity, the section has been reworded as follows (Line 298): The size of each constant depth pairwise subtable table determines the lookup table to match against for precomputed log-likelihoods. In the context of Rhometa and aligned reads, the number of genomes parameter used to generate lookup tables is instead taken as depth. Where the bam file is of a high depth, this can result in pairwise subtables of high depth. In such cases, available lookup tables may not be of high enough depth to cover them. A subsampling feature is included that is able to automatically downsample the BAM to a given depth.

2.15 Equation 1 is unclear. I think your \underline{t} notation is misleading? (Its described as a mean distance so I think so?) Underline would normally indicate a vector, overline for mean but since it is a parameter I don't think that is appropriate either.

Taking into consideration the reviewer's feedback, we have now removed the overline to avoid confusion.

2.16 Line 261 "we use the matched likelihood values for recombination rates" what does this specifically mean? Generally this paragraph is hard to parse.

We agree with the reviewer that the paragraph could be clearer. The paragraph in question has been updated for clarity as follows (Line 341): Next, we bring together the information we have generated thus far: the output of the lookup table and depth step and calculating r_{ij} step. For each variant site pair, we use the corresponding negative log-likelihood values from

the lookup table and depth step and on these apply linear interpolation to determine the negative log-likelihood values for r_{ij} value for that variant pair configuration. The interpolation is performed against the range of population recombination rates used to generate the lookup tables. This process is done for all the variant site pairs and the results final negative log-likelihoods for a given range of population recombination rates being evaluated, which again is typically between 0 - 100.

2.17 Line 269 "we introduce a novel weighting algorithm" you haven't motivated this specific form for the weighting. Does it approximate some sensible procedure, like a likelihood interpretation, or the standard deviation of the estimate for the corresponding normal normal distribution approximation? Does it appear elsewhere? Why this form?

Taking in consideration the reviewer's concern, this section of the method has now been updated for greater clarity with rationale for why this form of weighting is used (Line 350): Prior to computing the final log-likelihood sums we weight adjust the negative log-likelihoods. As the number of observations provided at a given depth represents the degree of evidential support towards the final prho estimate, we introduce a novel weighting algorithm that accounts for the additional information in high depth, high observation count site pairs relative to low depth / low count site pairs.The weighting algorithm is a simple solution to reliably add more weight/bias towards higher depth regions and the number of observations at that depth (there can be many regions of same depth). The weighting and final negative log-likelihood summation algorithms are as follows:

$$w_d(\rho) = \ln\left(\frac{\exp T_d}{\sum_\rho \exp T_d} dn_d\right) \quad \text{Equation 2}$$

$$\rho_{\max} = \operatorname{argmax} \sum_d w_d(\rho) \quad \text{Equation 3}$$

Here, d represents depths observed in the dataset. Weighting is performed on each per-depth table denoted by $w_d(\rho)$ (equation 2). In the right side of the equation 2, T_d is the unweighted per-depth table and n_d the number of unique variant site pairs. The reweighted negative log-likelihoods $w_d(\rho)$ are collected across depths and summed with respect to the range of population recombination rates being evaluated. The maximum negative log-likelihood value (closest to 0) then corresponds to the final population recombination rate ρ_{max} (equation 3) estimate.

2.18 "maximum log likelihood value (closest to 0) corresponds to the most likely final estimated population recombination rate" Firstly, can we get it clear whether this is a likelihood or not (perhaps a negative log-likelihood?) Secondly, "most likely final estimated" is very unclear.

In response to the reviewer's concerns, we have now renamed likelihood to negative log-likelihood throughout the manuscript, where applicable. This paragraph has also been significantly changed in response to the previous point which also addresses the second issue.

2.19 (Line 278 what happened to line numbers here?)

The issue with the line numbers has now been corrected.

2.20 Minor typography note: Throughout we've labelled "Equation X" but referred to them in text as "equation"

We have now changed instances of "equation" to "equation X" where applicable.

2.21 Figure 1: I'm not quite sure how to map the content to the text. For example, "Estimate theta (Python)" does not tell me whether this is the Wattersons Theta estimate. I think it is the (new?) Python components that need clarification, but you might introduce labels for all components. (I found that in the description L309, but the criticism still stands in trms of clarity.)

We apologise for the lack of clarity, the figure has been updated as follows::

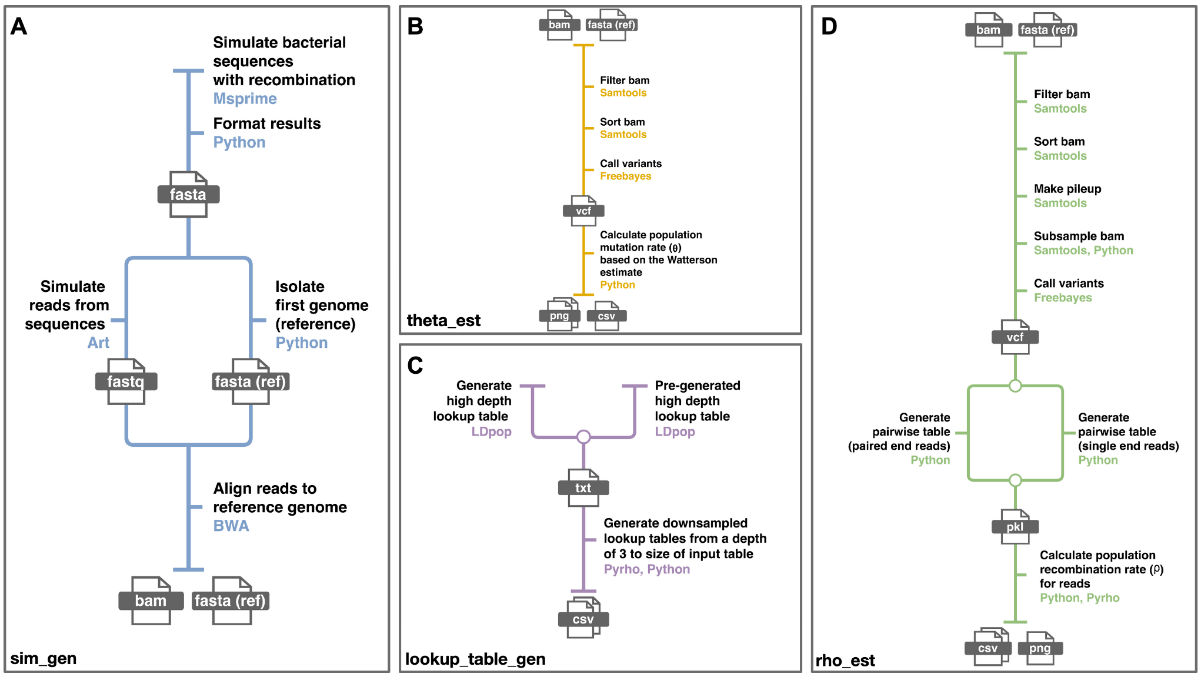

2.22 Line 362: "population_size was 1" what scaling is this? Does it mean something?

We have now reworded it to "effective population size" throughout.

2.23 Line 366: "The recombination events were simulated first then mutation events were simulated on top" - I assume this is referring to the method that msprime uses for neutral mutations. Either explain properly, or omit as it is standard.

In agreement with the reviewer we have opted to remove this sentence.

2.24 Line 367 Notation: "calculated as such"

*Following the reviewer's feedback notations are now used (Line 478): calculated as such: 2 * $i * N_e * u$. Generally notions are now used where applicable.*

2.25 Line 373 very verbose and not particularly clear or precise.

We agree with the reviewer and have now changed genome size to genome length throughout the document, which we believe was causing the confusion.

2.26 Line 384: Can we have a simple version here, and relegate the details to the methods?
We feel this information is important, but agree that it can be relegated to methods. We have therefore moved all of the information related to the datasets used for evaluation to the end of the methods section.

2.27 Para at Line 364: Typeset the equations.

We have used notations for equations as the reviewer has suggested.

2.28 The text contains the text "rho" and "theta" instead of the symbols throughout.

We have now changed these parameters to symbols throughout.

2.29 L437 "385 is the number that is thought to be false positives" how was this determined? If this number comes from elsewhere, just give the equation as Expected true variants/Total genome length.

We agree with the reviewer that the source of this could be more clear. This information comes directly from the Croucher et al paper. An exact quote is now provided in the manuscript for clarity as follows (Line 545) " We estimated the substitution probability between the donor and recipient and found it to be (17534-385)/2221315=0.00772, where 2221315 is the recipient genome length and based on the information provided by Croucher et. al (2012) "... the donor DNA identified 17,534 SNPs when aligned to the recipient sequence, of which 385 appeared to be false positives ... These positions were excluded from subsequent analyses." [37]."

2.30 Another way of putting the simulated Rhometa results is that regardless of the number of genomes, \rho is biased. This can be ok, but be up front with it.

In response to this point, the related paragraph in the discussion has been updated as follows (Line 944): "We then evaluated the performance of Rhometa on simulated datasets and the results (Fig 3 and 4) demonstrated that the read-based pipeline performs well and consequently represents a successful implementation of the composite likelihood population recombination rate estimator for metagenomic read-based datasets. As with LDhat and Rhometa_full_genome, the performance of the read based pipeline improves with the number of genomes present, having 80 genomes or more produces the best results. However, it should be noted regardless of the number of genomes there is a bias towards underestimation of ρ, though this is small when there are many genomes present (Fig 4) .

Very small ρ values, those between 0-1 (S3 Fig), are an exception as the implementation has a tendency towards over estimation.”

2.31 Line 506-514 Don't just tell us the numbers in the table. Either leave them in the table, or interpret them.

We agree with the reviewer and have removed this redundant text.

The following information has also been added (Line 785): seed averaged values were taken for the final results, refer to S1 table for individual seed results.

2.32 Line 518-525: Do you really mean that your estimate differs from mcorr by a factor of 2000? Or is there some scaling going on? I don't understand where the r/m=380 estimate in the next paragraph comes from, is this not their estimate?

We hope the following information provides clarity around this point:

The r/m of 380 is what we expect the r/m to be for the 84 sequences dataset based on the information provided in the Croucher et al paper. 380 is a lower bound estimate, so at the very least mcorr should be estimating this value, instead mcorr estimates 0.93, suggesting there was more mutation relative to the recombination, which cannot be so for this experiment. The actual r/m value should be greater than 380 since a region can experience multiple recombination events, which is reflected in the results of Rhometa with a value of 1922.97. Therefore we believe that Rhometa’s estimate is closer to the true r/m.

37. Line 527 You've given a very long paragraph containing many estimates and I don't understand the sources of the data for any of them properly.

We agree the paragraph could be improved and have therefore updated the paragraph for clarity as follows (Line 788):

*We can calculate the expected average r/m for a genome, as follows. In the Croucher et al paper [37], they state that the mean proportion in the recipient genome changed due to recombination was 1.4% using which we can estimate the average number of bases changed by recombination in a single genome as: 2221315 (recipient genome length) * 0.014 * 0.00772 (substitution probability as calculated in methods above) = 240.08. Also, in the paper it is stated that there were 2,312 polymorphic sites, 59 of which not coming from the donor, 6 of these sites were false positives, with the others likely being de novo point mutations or intragenomic recombinations. So we can take the upper bound for de novo mutations to be 53, then to get the average number of de novo mutations per genome we can divide by 84, which is the total number of sequences in the combined 5 and 500 ng dataset, 53/84=0.631.*

Using this information derived for the average number of bases changed by recombination and mutation in a single genome, we can calculate the expected average r/m for a genome as 240/0.631 = 380.3, for the combined 84 sequence dataset. The actual value should be higher due to the fact that a region can experience multiple recombination events, so this would be a lower bound estimate. With Rhometa we do observe a value greater than 380.3 of 1922.97 for the 84 sequence dataset.

2.33 Line 606(and much of this paragraph) is far too technical for a discussion. The purpose of this paragraph does not come through; it's just more results without context.

The paragraph in question has been reworked with an emphasis on how Rhometa produces more accurate results than mcorr. In re-working this lot of technical detail has been simplified.

The reworked paragraph (Line 968): Rhometa was further applied to a S.pneumoniae transformation experiment [37], where the extent of recombination could be directly quantified. This dataset was also analysed by the authors of mcorr for their paper, enabling a cross comparison between the two programs. Preparing and analysing the dataset (table 2, 84 sequences) in a manner performed by mcorr, it was found that Rhometa was able to accurately detect the higher rate of recombination relative to mutation as was expected for the dataset. From direct evidence, based on the information provided in the transformation experiment paper, we calculate a conservative lower bound for the ratio of recombination to mutation as r/m > 380.3, with the true value being much higher for the dataset analysed. Rhometa was able to meet this condition by estimating a r/m value of 1922.97 while mcorr estimated a r/m value of 0.93, which suggests that mutation was higher than recombination, which is extremely unlikely.

Reviewer #3: The paper is very well written and contains some interesting ideas for estimating recombination rates in microbial populations, however, I have a few concerns regarding the approach and validation.

3.1 The authors have claimed that: "The best way to study recombination in a microbial population is via metagenomics, which allows us to study microbes in their natural environment via direct sequencing and analysis of environmental DNA [7,8]"
The phrasing of this sentence is misleading to a reader because both [7,8] do not discuss microbial recombination. This also leads me to question the general claim: 'microbial recombination is best studied using metagenomics'; is it (currently) well supported by the literature?

We apologise for the lack of clarity here. We intended to say that metagenomics is the best way to study microbial processes in the environment in general. And we acknowledge that the inclusion of the word recombination complicates things. We have now removed the word recombination to clarify this point.

The line has now been updated as follows (Line 100):

"Currently the most viable way to study microbial populations is via metagenomics, which allows us to study microbes in their natural environment via direct sequencing and analysis of environmental DNA [7,8]. "

3.2 Additionally, I am (personally) a bit skeptical about this idea in general. Apart from the obvious complexities associated with shotgun metagenomic data such as the mixing of data from multiple sources, based on the literature, metagenome assembled genomes (MAGs) and metagenomic data in general can also suffer from serious data quality issues (for example, see https://doi.org/10.1093/bib/bbac413 and https://doi.org/10.1093/gbe/evaa238).

While overcoming such data quality issues is not the scope of this paper, how would Rhometa behave in the face of these concerns? How reliable will the recombination estimates be? In particular, given the fact that a metagenomic dataset will most likely contain a mixture of data from sources undergoing different evolutionary processes governing recombination, what exactly would a point estimate for recombination represent? Wouldn't a method that uses processed, carefully curated and filtered data be better suited for estimating microbial recombination? The authors should clearly address this point.

We wish to clarify that Rhometa does not make use of MAGs, which the papers mentioned focus on. This is an important advantage of Rhometa as the preparation of MAGs often lead to chimeric sequences that are not reflective of reality. Especially for the case of estimating recombination rates such chimeric sequences can give false results. Rhometa works with raw reads and the program includes filtering steps to account for quality issues that may exist.

The simulated read datasets were generated using ART which mimics real sequencing processes with empirical error models for illumina based systems and on these datasets we are able to get good estimates. Furthermore Rhometa has been tested on real reads from the s.pneumonia transformation experiment and we are getting good estimates. The program also worked on the Port Hacking dataset.

As such we feel Rhometa lays a strong foundation for recombination rate estimation with metagenomic read datasets.

3.3 Although Rhometa is designed to be used with metagenomic data, the S. pneumo dataset used for its validation is not a metagenomic dataset and the reference ATCC700669 (NC_011900.1) is not a MAG. The authors must have used this dataset for direct comparison with mcorr. However, I feel that Rhometa should be validated using real metagenomic data. I will be very interested to see how Rhometa behaves when faced with real-world complexities and potential data quality issues that I mentioned in my previous comment.

The reviewer makes a fair point, and in response we have now analysed real metagenomic datasets and demonstrated that Rhometa is indeed applicable to such datasets. To perform this analysis we selected published ocean metagenomic samples from Port Hacking, Sydney, Australia, on these datasets we performed long read assembly and identified contigs, MMseqs2 was then used for species identification and the reads were then aligned to these contigs. On the resulting bam files ρ estimations were performed. In this manner we were able to verify that Rhometa does work on real datasets.

Please see the new sections in the Methods and Results titled as follows, respectively: Real Datasets - Ocean Metagenomic Dataset and Evaluation on Real Datasets - Ocean Metagenomic Dataset.

3.4 Since there is limited availability of such experimentally estimated recombination rates for published metagenomic datasets, I think the authors can instead follow a cross-validation process to benchmark Rhometa. Although we will have no means to judge the prediction accuracy, assuming Rhometa works well in the presence of aforementioned challenges, the repeated estimates should have high precision.

We thank the reviewer for their suggestion, however, there are some potential pitfalls with trying to use cross-validation to understand predictive accuracy on real datasets, such as the presence of systematic bias or true variation existing among partitions of the dataset.

Simulated datasets are a great method for benchmarking Rhometa as the ground truth is known and is based on well established and verifiable methods such msprime. From which we know the program works well provided enough genomes are present.

In addition to the existing simulation studies we have we have expanded on the range of simulation studies and parameters evaluated to include mcorr and more scenarios, as follows:

Evaluating the changes in tract length used for analysis (Line 1070):

"In our tests with simulated datasets where the tract length was varied, it was found that misspecifying the tract length tends to linearly scale the final estimates in a predictable manner (S6 Fig). If a fixed value of tract length is given by the user for all analyses (i.e. an accurate sample-specific tract length is not provided by the user), then differences in inferred rho among samples could be due to either a change in the recombination rate or a change in tract length. The user will need to take this into account when interpreting the results.For instance, if performing same species comparisons across time and location, the tract length can be fixed to determine relative changes in the magnitude of recombination."

Evaluating the changes in θ used for analysis:
(Line 1095) "Generation of lookup tables require specification of θ , in our tests we have found that Rhometa is fairly robust to misspecifications of θ. It is only extreme misspecifications, such as misspecifications by a factor of 10, that seem to have a large impact (S5 fig)."

mcorr simulation sweep study (Line 908):

"Furthermore, mcorr was evaluated on simulated datasets used to test for Rhometa (S7 fig) where we expected to see an increase in the ϕ_pool estimated as the simulated ρ increases. However, the results showed no correlation between the recombination rate inferred by mcorr and the true simulated rate. Additionally, ϕ_pool, the value for recombination estimated by mcorr complicates interpretability by not using the standard parameter for population recombination (ρ). mcorr's limitation of looking at coding regions provided in a gene annotation file again complicates analysis as this information is not always available. Rhometa builds on established methods and theoretical foundations, without the need for gene annotation files and based on our testing, produces accurate results."

3.5 Additionally, the simulated data used to validate RhoMeta are also completely 'noise-free' and not technically 'metagenomic'. In fact, the entire simulated data validation pipeline seems far too simplistic for a tool designed for metagenomic data. Additionally, I am not sure if msprime is the best tool to simulate data for this purpose at all, for example, see section 2.2. of https://doi.org/10.1093/bioinformatics/bty630 for a list of tools that might be better

suited for this purpose. In addition, the authors should also properly benchmark and report performance metrics (CPU, RAM requirements, etc.), including its limitations in terms of maximum genome size, maximum number of sequences, etc. (as applicable).

We wish to clarify that Mspirme is different to insilicoseq in that it is used to generate sequences with fixed recombination, mutation and other factors. Insilicoseq then uses these sequences to generate reads, however we are already performing this step with ART (Line 489) " ART [35], which simulates sequencing reads by mimicking real sequencing processes with empirical error models. The reads were simulated based on the illumina HiSeqX TruSeq system" . Using uniform distribution in insilicoseq, the results would be no different.

Regarding performance metrics, the following benchmark was performed and details added to the manuscript (Line 824): The 84 sequence dataset was also used to benchmark Rhometa. The bam file for this dataset is 11.42 GB in size. On a system with 4 CPUs and 32 GB the rho_est pipeline took 1h 45m to complete and the theta_est pipeline took 2h 45m to complete. Also the following line (Line 74), "The program performs well on large BAM files and the included subsampling functionality ensures that files of arbitrarily large size are subsampled and analysed." Talks about how very large files can be processed.

3.6 Finally, the inability to infer the recombination tract length could be a potentially major pitfall. Firstly, the recombination estimate seems to depend on this value, and secondly, since Rhometa is intended to be used with metagenomic data, a potential user will find it nearly impossible to rely on the previous literature to find a 'guess' for this parameter. Therefore, the authors should clearly outline a process to estimate or guess this value.

We understand the reviewer's concern and have performed simulation studies around misspecifications of the tract length to help the user get a clearer understanding of it's impact and provide an example on how the user can work around this limitation (Line 1070):

"In our tests with simulated datasets where the tract length was varied, it was found that misspecifying the tract length tends to linearly scale the final estimates in a predictable manner (S6 Fig). If a fixed value of tract length is given by the user for all analyses (i.e. an accurate sample-specific tract length is not provided by the user), then differences in inferred rho among samples could be due to either a change in the recombination rate or a change in tract length. The user will need to take this into account when interpreting the results.For instance, if performing same species comparisons across time and location, the tract length can be fixed to determine relative changes in the magnitude of recombination."
